# Zooplankton Community Profiling in a Eutrophic Freshwater Ecosystem-Lake Tai Basin by DNA Metabarcoding

**DOI:** 10.1038/s41598-017-01808-y

**Published:** 2017-05-11

**Authors:** Jianghua Yang, Xiaowei Zhang, Yuwei Xie, Chao Song, Yong Zhang, Hongxia Yu, G. Allen Burton

**Affiliations:** 1 State Key Laboratory of Pollution Control and Resource Reuse, School of the Environment, Nanjing University, Nanjing, Jiangsu 210023 China; 2 Jiangsu Environmental Monitoring Center, Nanjing, Jiangsu 210000 China; 3 School of Natural Resources and Environment, University of Michigan, Ann Arbor, Michigan USA

## Abstract

Communities of zooplankton, a critical portion of aquatic ecosystems, can be adversely affected by contamination resulting from human activities. Understanding the influence of environmental change on zooplankton communities under field-conditions is hindered by traditional labor-intensive approaches that are prone to taxonomic and enumeration mistakes. Here, metabarcoding of cytochrome c oxidase I (COI) region of mitochondrial DNA was used to characterize the genetic diversity of zooplankton. The species composition of zooplankton communities determined by metabarcoding was consistent with the results based on the traditional morphological approach. The spatial distribution of common species (frequency of occurrence >10 samples) by metabarcoding exhibited good agreement with morphological data. Furthermore, metabarcoding can clearly distinguish the composition of the zooplankton community between lake and river ecosystems. In general, rotifers were more abundant in riverine environments than lakes and reservoirs. Finally, the sequence read number of different taxonomic groups using metabarcoding was positively correlated with the zooplankton biomass inferred by density and body length of zooplankton. Overall, the utility of metabarcoding for taxonomic profiling of zooplankton communities was validated by the morphology-based method on a large ecological scale. Metabarcoding of COI could be a powerful and efficient biomonitoring tool to protect local aquatic ecosystems.

## Introduction

Global freshwater and wetland ecosystems face multiple threats to their stability, including changes in land use, nutrient and toxicant pollution, and climate change^1, 2^. These disturbances could impair natural functioning (e.g. nitrogen cycle) and alter the structure (e.g. species composition) and function of communities and ecosystems. One of the significant outcomes of global environmental changes caused by activities of humans is greater concentrations of nitrogen and phosphorous in aquatic ecosystems. Due to overuse of fertilizers in agriculture and discharge of wastewater effluents to freshwater rivers and lakes, eutrophication leads to loss of biodiversity and deterioration of water quality^3–5^.

Biodiversity of aquatic ecosystems has enormous economic and aesthetic value and is largely responsible for maintaining and supporting environmental health and ecosystem services^6^. Zooplankton play vital roles in biogeochemical cycling of C and N, and aid the stability of aquatic food webs^7^. In addition, many zooplankton are sensitive to external perturbations and, consequently, a useful indicator of environmental stressors such as climate change, chemical and organic pollution^8–10^. However, understanding the influence of environmental change on zooplankton communities is hindered by the traditional taxonomy challenges^11, 12^. Traditional methods of identifying species, and enumerating individuals based on morphology is costly, time-consuming and requires highly trained individuals with expertise in identifying species in zooplankton communities, especially in large scale environmental investigations and monitoring programs^13^. In addition, traditional biological monitoring is only feasible for easily observable species, however, it is difficult or virtually impossible to use morphology-based method for some taxonomic groups (e.g. larvae of copepoda).

Metabarcoding technology, which can be used to characterize compositions of species by use of environmental DNA and next generation sequencing (NGS)^14–16^. In particular, metabarcoding have found several applications^17^, such as investigating biological diversity^18^, characterizing prey composition in gut contents^19^, and analyzing food-web dynamics^20^. Many studies have demonstrated that metabarcoding improve taxonomic resolution and can be useful in assessing biodiversity of zooplankton^11, 12, 19^. Several gene regions have been used in metabarcoding of zooplankton assemblages, such as hypervariable regions of 18S rRNA^12, 21, 22^, 28S rRNA^23^, mitochondrial 16S rRNA^24^ and COI^25^. Mitochondrial COI is one of the most commonly sequenced regions for biodiversity analyses of animals, including zooplankton diversity^26^. However, the standard COI primers target the 658 base pair (bp) barcoding region, whose size is considered too large for high throughput sequencing platform (e.g. Ion torrent PGM). Leray *et al*. designed a new COI primer set which targets a 313 bp fragment to characterize the gut contents of fish^19^. Although the target length of this primer is very suitable for NGS and the products have a good performance on species identification, no study available uses it to characterize zooplankton diversity from the environmental samples. In the present study, metabarcoding of mitochondrial COI 313 bp region of mtDNA was used to characterize the genetic diversity of zooplankton in a eutrophic freshwater ecosystem - Tai Lake Basin in China. The specific objectives include: 1) to develop a metabarcoding protocol to assess the components and structure of freshwater zooplankton communities; 2) to validate the results of metabarcoding by morphology-based monitoring data.

## Results

### Profile of zooplankton composition based on metabarcoding data

**Figure 1:**
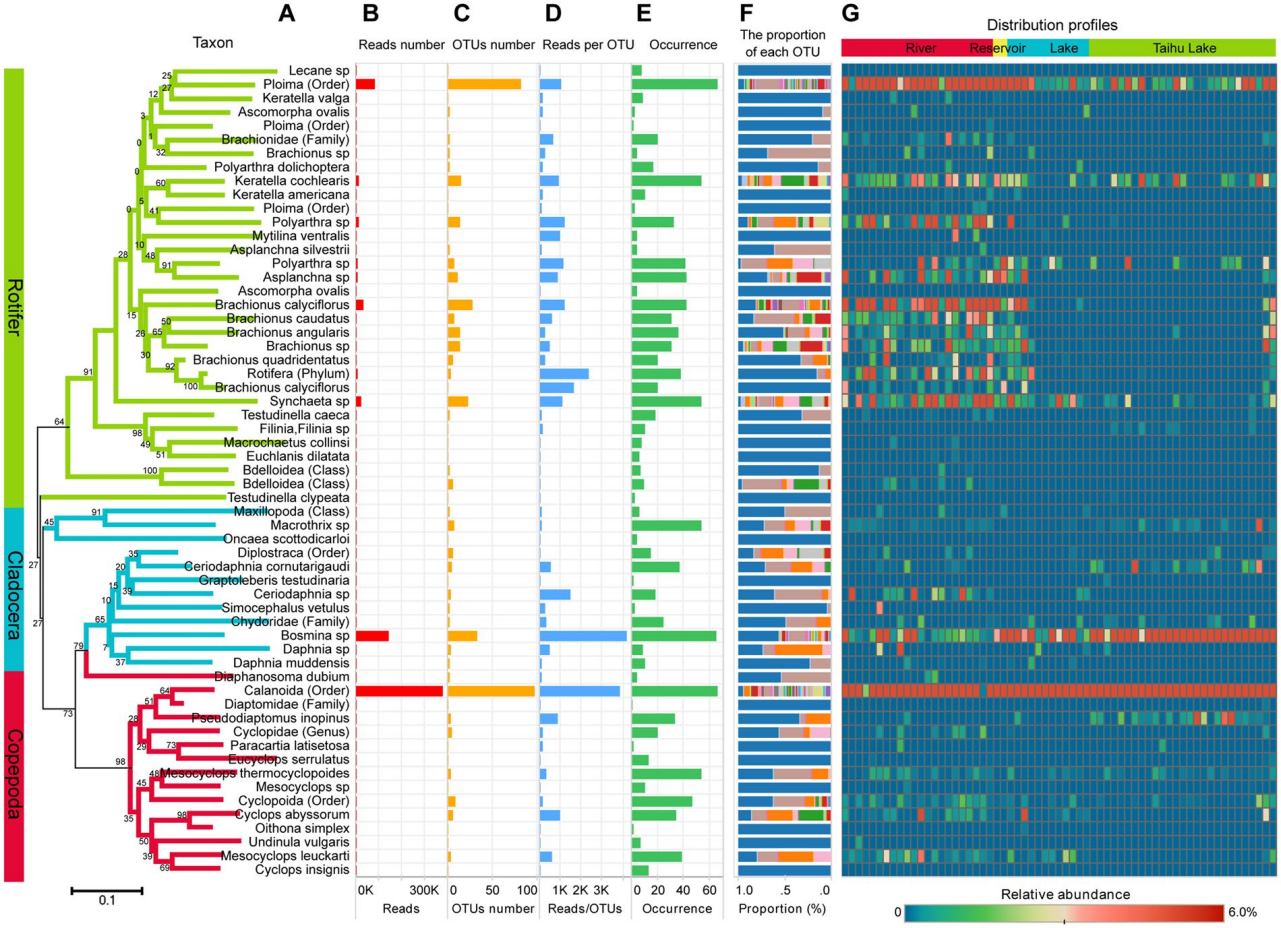
Phylogenetic distribution of assignable components of zooplankton CO1 diversity. (**A**) A tree diagram of representative sequence of each taxon. Distance was measured by the number of base substitutions per site, based on the Kimura two-parameter (K2P) method. One thousand bootstrap trials were run using the neighbor-joining algorithm of the Mega 6.0 program. (**B**) Number of reads of each taxon. (**C**) Number of OTUs of each taxon. (**D**) Number of reads per OTU. (**E**) Occurrences of each taxon in all samples. (**F**) Proportions of OTUs in the same taxon. Different color represents different OTU, and stacked bar means relative abundance of each OTU. (**G**) Distribution of each taxon in all samples. The color weighted by relative reads abundance (e.g. 0.01 means the number of reads for this taxon accounts for 1% of total number of reads).

DNA metabarcoding of zooplankton communities collected by the plankton net provided a reliable community profile on the 69 different sites across the Tai Lake basin sampled from 28-11-2013 to 12-12-2013 (Fig. S1 and Table S1). Most sequences (910,741 in 950,283, 95.8%) of eukaryotic mitochondria CO1 obtained can be assigned to a specific taxon (sequence similarity >80% and posterior probability >60% in SAP) by using NCBI Genbank and local species database and about 90% (819,253 in 910,741) of the sequenced reads belong to zooplankton individuals (Fig. S1). Rotifer, Copepod and Cladocera represented the three major taxa (Fig. S1, Fig. 1). There were five taxa with large numbers of reads, including Ploima (order), *Branchionus calyciflorus*, *Synchaeta sp*, *Keratella cochlearis*, *Bosmina sp*, and Calanoida (order). In general, the taxa that had a highly number of reads were also found from many samples. However, there were also some species, such as *Pseudodiaptomus inopinus*, *Mesocyclops thermocyclopoides* and *Mesocyclops thermocyclopoides* which contained only several hundred reads but were detected in many samples.

### Components of eukaryotic plankton biodiversity

Approximately 22% (217 in 1003) of OTUs observed in this study did not match any reference barcode or match to the “environmental invertebrate sample” in the NCBI database and were labelled as “unassigned”. The unassignable OTUs represented 7.6% of total reads. Approximately 40% of total assigned OTUs were classified as rotifers, while the greatest proportion of read counts were classified as calanoida. Only a small proportion of OTUs and reads were classified as Cyclopoda species. Due to the universality of eukaryotic primers used, approximately 5% of barcodes were classified as diatoms. The Neighbor-Joining (NJ) tree diagram based on the CO1 sequences demonstrates that all zooplankton taxa were clearly divided into three subgroups (rotifer, cladocera or copepod) (Fig. 1). Most rotifers had greater occurrences rivers, than in Tai lake or other small lakes. Nevertheless, *Keratella cochlearis* and Ploima (Order) are detected in both rivers and lakes. Although *Bosmina sp*. was observed in almost all samples, the relative abundance in lakes was greater than it in rivers (Fig. 1). The depth of sequencing (10000 reads per sample) is enough to saturate the sampling curve for identification of zooplankton communities (see SI, Fig. S2).

### Comparison of metabarcoding data with monitoring data based on morphology

**Figure 2:**
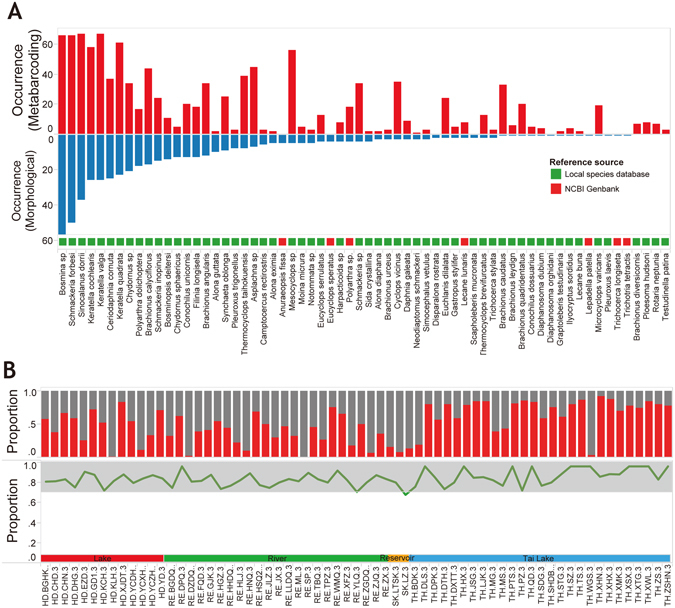
Comparison of metabarcoding data with traditional monitoring data on zooplankton species monitoring. (**A**) Frequency of occurrence of each species by metabarcoding and morphological method. (**B**) The top panel shows the proportion of OTUs in metabarcoding data matched to the morphological identification at each site. Red color indicates the proportion of species detected by both morphological method and metabarcoding. Grey color indicates the proportion of species detected by metabarcoding only. The bottom panel shows the proportion of species detected by metabarcoding at each sample. (e.g. 0.8 means 80% morphological species detected by metabarcoding).

**Figure 3:**
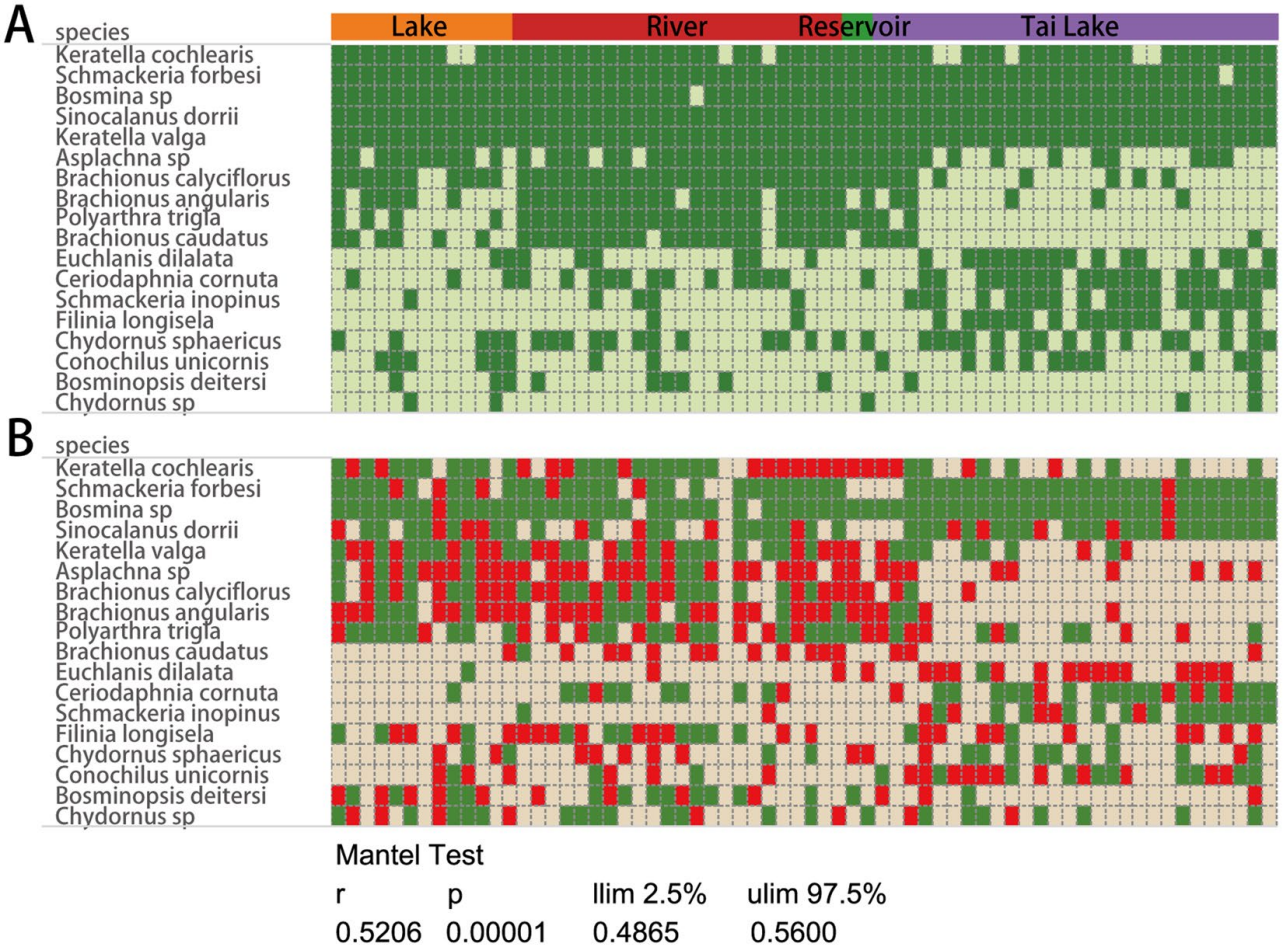
Comparison of the distribution characteristics of common species between metabarcoding and morphological data. Common species means the frequency of occurrence >10 samples. (**A**) Metabarcoding data. Green dot indicates the species were detected by metabarcoding. (**B**) Morphology monitoring data. Green dot indicates the species appeared in both quantitative and qualitative sample and red dot indicates the species only appeared in the qualitative sample. (R = 0.52, p = 0.0001, mantel test, 9999 permutations). “llim” means empirical upper confidence limits of r value. “ulim” means empirical lower confidence limits of r value.

**Figure 4:**
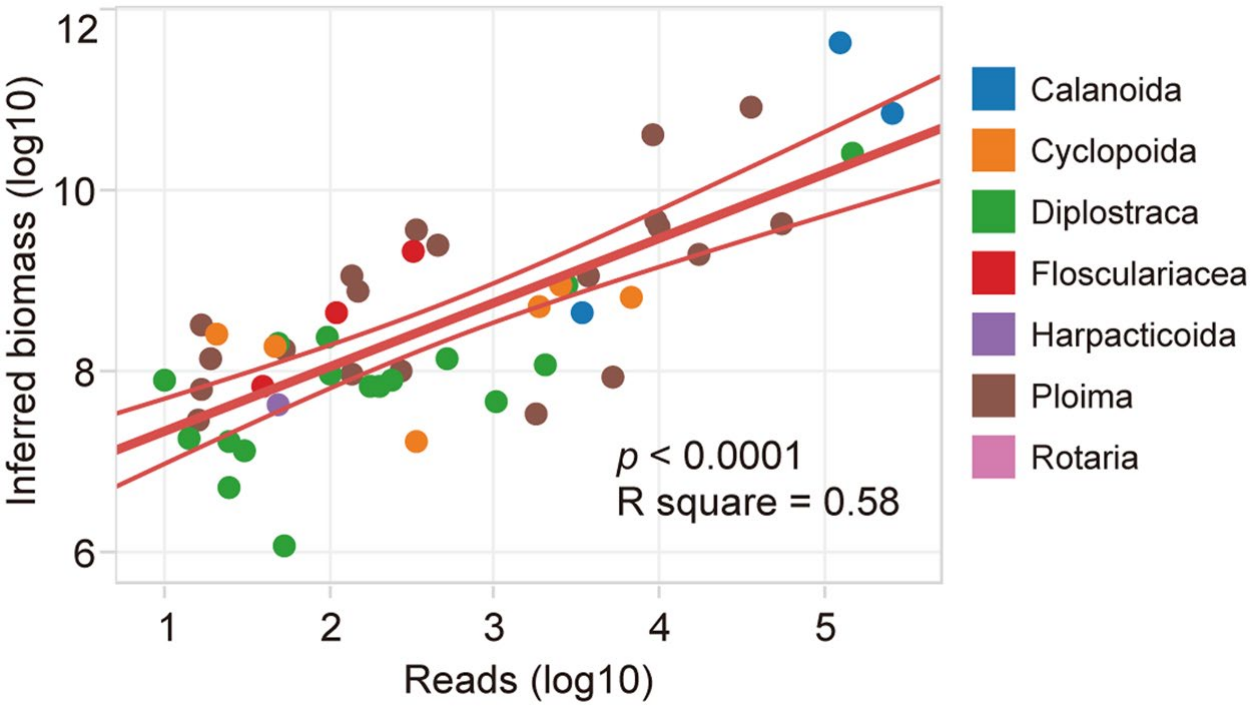
Relationship between inferred biomass of zooplankton in traditional monitoring data and the number of reads in metabarcoding data. The inferred biomass of zooplankton was roughly estimated by the density and body length (biomass = density × bodylength^3^).

The profile of zooplankton communities from metabarcoding data is consistent with the results of morphology based data. There were 68 species of zooplankton that were identified based on morphology and 52 of those were also detected by use of DNA metabarcoding (see SI, Fig. S3). Most of the missed species (those species that were not found in metabarcoding but were in morphological assessments) have only a few or no reference barcode sequence in Genbank (see SI, Figs S4 and S5). Second, the occurrence frequency of zooplankton in metabarcoding data were also consistent with traditional morphological identifications (Fig. 2A). In addition, about 80% of species that were identified by the morphological method could be detected by metabarcoding in most samples (Fig. 2B). Finally, the distribution characteristics of common species (frequency of occurrence >10 samples) by metabarcoding also exhibited good agreement (R = 0.52, p = 0.0001, mantel test with nperm = 9999) with the morphological monitoring data (Fig. 3). The comparison of zooplankton inferred biomass (inferred biomass = density × bodylength^3^) with the NGS read counts demonstrated that, as a new monitoring tool, metabarcoding can work well for zooplankton investigations and the monitoring results were highly consistent with the data based on traditional, visual identification based on morphology (Fig. 4).

16 species identified by morphology were not detected by metabarcoding, most of which are not commonly observed in the aquatic ecosystems of Lake Tai basin (see SI, Fig. S3). In addition, only 3 species of the 16 had CO1 barcode sequences in the NCBI Genbank (see SI, Fig. S4).

There were 20 species identified by morphology that appeared in more than ten samples. Eighteen of the 20 species could be detected successfully by metabarcoding, except two species, *Gastropus minor* and *Keratella ticinensis*. Both species have no barcode sequence available indicated that the sequence from the missing species might not be annotated to the species level. In addition, a lot of rotifer OTUs cannot be assigned to the species level. For example, both ploima (order) and rotifer (phylum) represented more than 50 OTUs and appeared in at least 30 samples, but just assigned to the order or a higher taxonomic level. A similar situation also exists in copepod and cladocera. Nevertheless, the metabarcoding method can detected more species than the traditional morphological method. Metabarcoding detected an additional 37 OTUs that were not found using morphology alone (Fig. S5).

Although rotifer with a small body size had a high density, larger copepods represented more of the inferred biomass overall (see SI, Fig. S6). In morphological monitoring data, the inferred biomass decreased from copepod, rotifer to cladocera. Consistent with the inferred biomass data, copepod, rotifer and cladocera read number from metabarcoding also decreased (Fig. S7). In addition, the number of reads in metabarcoding has a highly positive correlation (Pearson correlation, *p* < 0.001) with zooplankton inferred biomass determined from density and body length (Fig. 4).

### Differences in zooplankton communities between lakes and rivers

**Figure 5:**
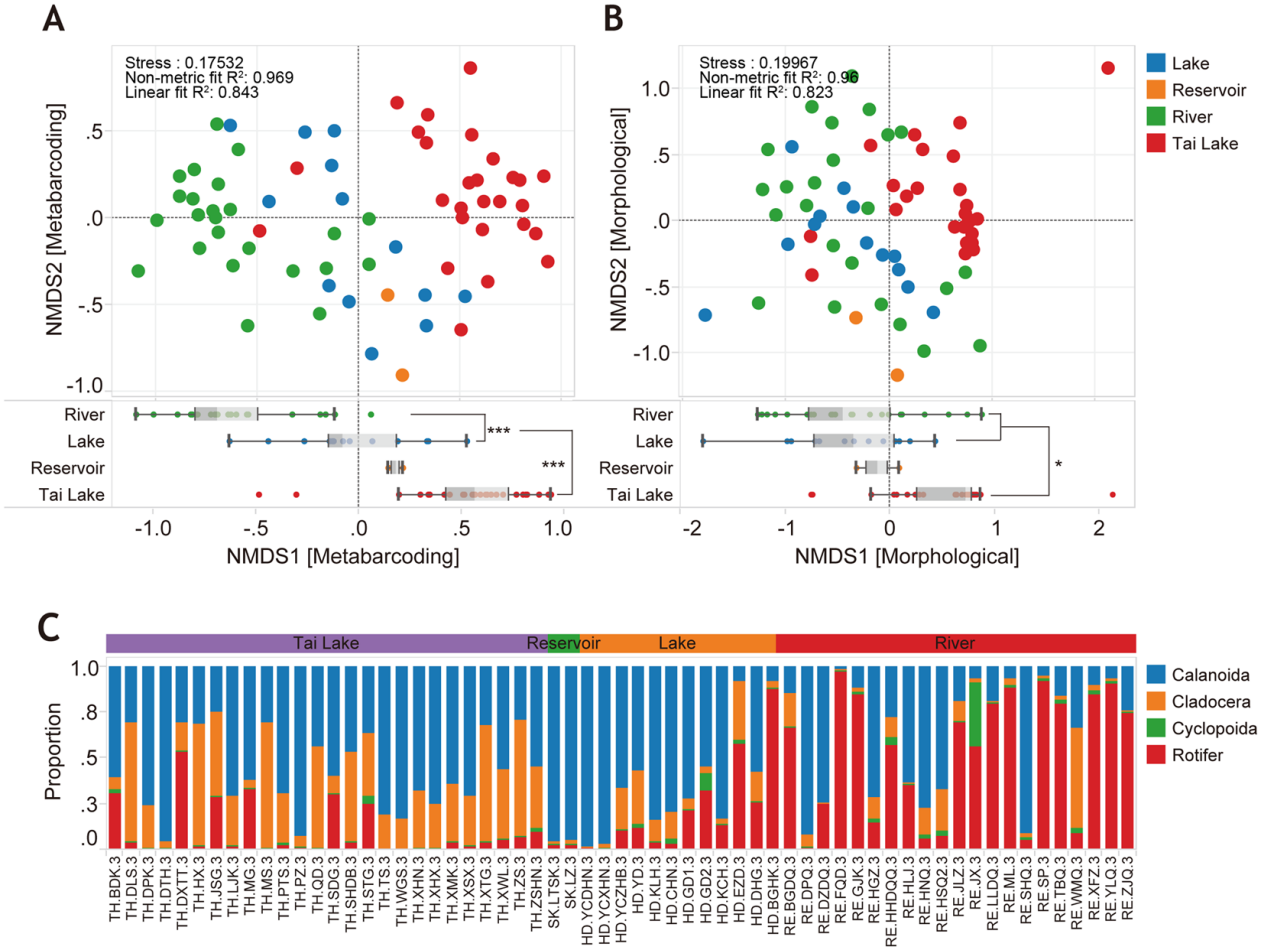
Composition of species and diversity of various types of aquatic environments (Tai Lake, Reservoir, River and Lake. Here, “Lake” means all the relative small lakes around Tai Lake). (**A**) Non-metric multidimensional scaling (NMDS) of zooplankton community components based on CO1 OTUs. (**B**) NMDS analysis of zooplankton community components based on morphological data. The boxplot below the NMDS illustrates the differences of NMDS1 between types of aquatic environments. Significance of ANOVA with Duncan’s post hoc tests was determined at *P* values < 0.001 (***) and <0.05 (*). (**C**) Proportions of rotifer, calanoida, cyclopoida and cladocera (based on metabarcoding data) in each sample.

The composition and biodiversity of zooplankton communities were dissimilar among different types of water bodies (Figs 1 and 5). NMDS analysis based on OTU read abundance indicated community samples collected from lakes could be clearly discriminated from rivers. In addition, samples from Tai Lake were discriminated from those collected from smaller lakes (Fig. 5A). Although the morphological data can also indicate the statistical difference of the zooplankton composition between Tai Lake and river/lake (*p* < 0.05), but morphological approach failed to discern the difference between rivers and small lakes (Fig. 5B). On the other hand, metabarcoding data showed that river, lake and Tai Lake had distinct zooplankton community profile (*p* < 0.001) in the NMDS1 by ANOVA with Duncan’s post hoc tests. Proportions of the three main groups (rotifer, copepod and cladocera) also differed among water body types. In samples from rivers, in general, most species of zooplankton were rotifers, which have relatively smaller bodies compared to copepods. However, the proportion of copepods was greater in lakes and rotifers were less common (Fig. 5C).

## Discussion

Compared with traditional morphology-based taxonomy, DNA metabarcoding identified a greater number of species and provided additional information to understand the ecology of zooplankton community. Since the zooplankton community was enriched by filtering the lake water with a plankton net, most of the sequences obtained in the present study were assigned to the zooplankton. Rotifera, cladocera and copepod, which are the three main zooplankton groups and the primary functional groups in freshwater aquatic ecosystems, can be detected by metabarcoding, although other aquatic organisms, including aquatic insects, diatoms, protista, and nematodes were also detected marginally (11.2% of zooplankton sequences) because of the universality of primers. Approximately 20% of OTUs, representing a small proportion (7.6%) of total reads, were unassignable, which suggested the rare and minute taxa that escaped previous characterization^27^. Species of rotifer contributed significantly to biodiversity of the freshwater ecosystems^28^ because rotifer OTUs represented more than half of the total OTUs in zooplankton communities. Calanoida, for which the greatest abundances of reads were observed, represented a large proportion of the biomass in the ecosystem. The identification of zooplankton species was significantly improved by metabarcoding, especially for those copepods at early life stage (traditional morphological method assigned it to “copepod larvae”). Taxonomy based on DNA barcoding makes it possible to identify the species of copepods larvae^29^, which improves the description of the species composition.

The inferred biomass of zooplankton estimated by density and body length had a good linear correlation with the read counts in metabarcoding (Fig. 4). The utility of DNA metabarcoding for quantifying the species abundances is currently limited by both biological and technical biases which influence sequence read counts, such as PCR amplification biases, primers biases, DNA copy number variation^30^. Even sequencing biases will affect the abundance estimation in metabarcoding analysis^31, 32^. For the first time, we tried to estimate the biomass of zooplankton using body length and density of zooplankton in the present study, and found that the inferred biomass showed a strong linear correlation with the number of sequence reads. Although most of the freshwater zooplankton are rarely homogenous in shape, body length parameter can basically reflect the size of zooplankton. So, the inferred biomass may be useful to estimate the abundance of zooplankton when the real biomass data (like dry weight) is absent. However, the relationship between inferred biomass and read number does not indicate that metabarcoding is an accurate quantitative biomonitoring tool for zooplankton communities. Because the read counts are highly dependent on the amount of DNA as well as gene copy number and primer bias^33^. We have shown that metabarcoding read counts can provide a preliminary or a semi-quantitative estimation of relative abundance with less effort than that based on enumeration of individuals and dry weight using traditional methods^34^. Overall, the metabarcoding of bulk DNA approach presented here provided a more complete characterization of the species composition of zooplankton, which may be applicable to other taxa (diatom, Protista, insect and even fish).

Structures of zooplankton communities were significantly different in lake and river ecosystems. In general, rotifers were more abundant in riverine environments than lakes and reservoirs. There are a number of reasons rotifer might be fewer in number in lakes (temperature, grazer pressure, food availability, hydrodynamics)^35–37^. Previous studies have illustrated that smaller-bodied zooplankton species, which are more tolerant of relatively high temperatures, have more rapid cycles of development and reproduction, than do larger species^38–40^. The smaller body size, phenotypic plasticity and adaptable trophus apparently likely contribute to success of rotifers^41^. The abundance of smaller-bodied zooplanktons (such as rotifers) were reduced in lakes suggest that the zooplankton community is likely more vulnerable to environmental stressors (e.g., temperature rise and eutrophication) than those in rivers. Since smaller zooplankton cycle more nutrients larger species^42^, this implies that nutrient cycling may also be reduced in lakes. In addition to size differences, biodiversity based on OTUs was greater in riverine than lake environments. Over the past two decades, it has been shown that ecosystems with greater biodiversity are more efficient at removing nutrients from water than are ecosystems with fewer species^43–45^. So the greater biodiversity of rivers suggests this community has adapted to cycling greater amounts of nutrients, thus maximizing use of available nutrients. The observed differences of community composition between water body types may be caused by many environmental factors including the differences in physical and hydrologic conditions^46^. Some environmental toxic factors, e.g. total ammonia nitrogen (TAN), also has a high potential to cause shifts in zooplankton communities^47^.

Although the morphological data can discriminate the difference of zooplankton composition between rivers and lake, such difference was more significantly shown by metabarcoding data. It indicated that metabarcoding can better reveal the composition characterized of zooplankton than morphological identification. And it is more sensitive to reflect the alteration of zooplankton community against environmental factors. Because metabarcoding significantly improved the capability of species identification. This is important for us to understanding the influence of water quality on zooplankton structure.

Although metabarcoding is a very promising approach for biomonitoring, there are still some limitations and weaknesses by itself. Firstly, some technical biases related to the PCR reaction, primer specificity and even sequence analysis would affect the final results^14, 48^. So multi-PCR reaction replicates and using different primers for different taxon are necessary to reduce the biases of PCR. Furthermore, oligonucleotides (capture probes) that target conserved regions or directly sequence the eDNA can also avoid PCR error and primer biases^49^. The standardized NGS data analysis pipeline is also important to improve the reliability of metabarcoding and reduces the divergences between molecular and morphological results^50, 51^. Secondly, the incompleteness and inaccuracy of reference databases were believed to be the main hindrance to assigning taxonomy to metabarcoding sequences and lead to some species to be unidentified^52^. Most of the species which have barcode sequence that can be assigned to the species level. So constantly improving reference databases, especially local species database, is important for routine biomonitoring. Lastly, DNA based approach could not distinguish the life stage and health status of the individuals. Therefore, if the information of life stage and health status is important, combining the molecular approach and morphological methods together for bio-assessment are recommended.

Preforming multi-marker genes might provide potential advantages in the future study of metabarcoding. The depth of sequencing provided by NGS makes this more feasible than the past. Multi genes (e.g. mitochondrial 16S rRNA and nuclear 18S rRNA) provide important confirmatory evidence for phylogenetic analysis. Many studies demonstrated that COI was an ideal candidate marker of animal for high-throughput sequencing-based biodiversity assessment. However, some taxa in aquatic communities, including dominant taxonomic groups such as Copepod and Cladocera, might be difficult to be PCR amplified when using COI for barcoding analysis^53^. Using different types of genetic markers could reduce the bias of primers and enhance the performance of biodiversity assessment in aquatic communities.

In summary, the genetic diversity of zooplankton was used to characterize the freshwater community on a large ecological scale, in Lake Tai basin of Eastern China. Metabarcoding of community DNA significantly increased the number of zooplankton taxa that can be observed in biomonitoring. The species composition of zooplankton community from metabarcoding data was consistent with the results based on traditional morphological data. DNA based approach could more clearly show the difference of zooplankton community between lake and river ecosystems. Finally, metabarcoding can provide a preliminary or even a semi-quantitative estimation of abundance with less effort than that based on enumeration of individuals and dry weight by traditional taxonomy. The developed metabarcoding protocol could be a powerful and efficient bio-assessment and biomonitoring tool to protect local aquatic ecosystem.

## Materials and Methods

### Sampling sites

The catchment of Tai Lake (Ch: *Taihu*), which is situated in the lower reaches of the Yangtze River in south-eastern China, is one of the most densely populated and developed areas in China^54^. About 17% of the total territory is covered by lakes and river channels. Tai Lake is the third largest freshwater lake in China (2400 km^2^). In the vicinity of Tai Lake, there are several small lakes, Ge Lake (146.9 km^2^), Yangcheng Lake (118.9 km^2^), Changdang Lake (89.0 km^2^), Cheng Lake (40.6 km^2^), Kuncheng Lake (17.9 km^2^), Yuandang Lake (13.0 km^2^), Kuilei Lake (6.73 km^2^), and Ezhendang Lake (5.2 km^2^). In the current study, a total of 69 sampling sites (27 sites in Tai Lake, 25 sites in the Yangtze River and tributaries, 15 sites in smaller lakes and 2 sites in reservoirs) across the basin were sampled from 28-11-2013 to 12-12-2013. Sampling sites were grouped into four categories according to the type of water body: 1) Tai Lake, 2) Reservoir, 3) River and 4) Lake. Here, “Lake” means all the relatively smaller lakes around Tai Lake (Fig. S1 and Table S1).

### Collection and identification of Zooplankton

Quantitative and qualitative sampling methods are the most commonly used standard sampling method for plankton diversity analysis. At each sampling site, two quantitative samples of zooplankton were collected by use of a plankton net (46-μm mesh, 0.316-m opening diameter) through which 20 L of water were passed (Fig. 1B). One sample was used for metabarcoding analysis while the other was used for traditional identification of zooplankton based on morphological taxonomy. A qualitative sample (zooplankton enriched by plankton net from a large volume of lake water) was collected to maximize the morphological identification of zooplankton at each site. In the laboratory, the zooplankton samples that enriched by plankton net from 20 L water were further filtered through 5-μm microporous filter paper (Millipore, USA). Filter membranes were then placed in a 5-mL centrifuge tube and stored at −20 °C until extraction of DNA. The E.Z.N.A. water DNA kit (Omega, USA) was used to isolate DNA from zooplankton trapped on the filter paper. All zooplankton were identified to species by use of traditional taxonomic methods^55–57^, based on morphology although in a few cases the specimens could be identified only to genus or higher level of taxonomy.

### Amplification by PCR and next generation sequencing (NGS)

The gene for eukaryotic mitochondria cytochrome c oxidase I (COI) was amplified by use of the degenerate primer (mlCOIintF and gHCO2198)^19^. Amplification by PCR was performed with a final volume of 50 μL, made up of 1 μL of 10 μM of primers, 2 μL of DNA template, 37.8 μL of ultrapure water, 5 μL of 10 × PCR High Fidelity PCR buffer, 2 μL of MgSO_4_ (50 mM), 1 μL of dNTP mix (10 mM) and 0.2 μL of Platinum Taq DNA polymerase (Invitrogen, USA). To minimize potential bias of the PCR, triplicate PCR reactions were performed for each sample. Amplifications by PCR were performed in 96-well plates using a SureCycler 8800 thermal cycler (Agilent Technologies, USA). Although a lower PCR cycle number can help improve the diversity of PCR amplicons from environmental samples^58^, a “touchdown” PCR profile with 41 cycles was used to maximize the products and minimize the probability of non-specific amplification because of the high level of degeneracy of the primer sequences^19^. PCR was conducted for 16 initial cycles as follows: denaturation for 10 s at 95 °C, annealing for 30 s at 62 °C (1 °C per cycle), and extension for 60 s at 72 °C, followed by 25 cycles at an annealing temperature of 46 °C. The final extension was performed at 72 °C for 10 min. A negative control reaction with no DNA template was included in all experiments. PCR products were detected on a 2% (w/v) agarose gel, and fragments from the gel were purified by use of the MinElute gel extraction kit (Qiagen, CA, USA). After purification on the gel, products of PCR were quantified by use of Qubit dsDNA HS assay kits (Invitrogen, USA), and the final concentration was adjusted to 10 ng/μL using molecular grade water.

To ensure a homogeneous number of sequencing reads from each sample, amplicons were mixed in equal concentrations (10 ng/μL) in an equimolar pool. One hundred ng of the pooled amplicon in a total volume of 79 μL of nuclease-free water was used in end-repair and ligation of adaptors by use of the Ion Plus fragment library kit (Life Technologies, USA) according to the manufacturer’s protocols. To eliminate primer dimers and PCR artifacts <100 bp, the end-repaired and ligated adaptor DNA was purified with the Agencourt AMPure XP kit (Beckman Coulter, Germany). The purified amplicon library was then transferred to new 1.5-mL Eppendorf LoBind tubes (Eppendorf, Germany) and assessed for region size distribution and DNA concentration using an Agilent 2100 bioanalyzer (Agilent Technologies, USA).

Quantified, size-selected amplicon libraries (467 bp), including amplification primers, MID tags, and Ion Torrent adaptors, were serially diluted to a final concentration of 100 pM and attached to the surface of Ion Sphere particles (ISPs) using the Ion PGM template OT2 400 kit (Life Technologies, USA). The ISPs were enriched on the Ion OneTouch enrichment system (Life Technologies, USA) and together with the template were sequenced on “318 v2” micro-chips using the Ion PGM sequencing 400 kit (Life Technologies, USA) with the Ion Torrent PGM (Life Technologies, USA) for 850 flows according to the manufacturer’s protocols.

### Bioinformatics

The ION Torrent server auto-sorts sequences into groups, based on the library barcode, then generates a FASTQ format file containing bases and quality information. Fastx toolkits and Bio-python were then used, to reverse complement the FASTQ file and to convert the FASTQ file to FASTA and qual files, respectively^59^. The QIIME (Quantitative Insights into Microbial Ecology v1.8.0) platform^60^ was used to filter low-quality reads and to discard reads with more than two mismatches in the primer sequence by the split_libraries.py command with the parameters –w 50 –s 20 –m 2. Chimera filtering were performed by “usearch -uchime_denovo” in USEARCH program which base on UCHIME algorithm^61^. The above steps were completed using the Bio-Linux 8 system, which integrates all of the above-mentioned tools^62^. Short reads (<250 bp) were filtered using the “Biostrings” package in R software (3.1.2) with the Bioconductor environment^63^. Operational taxonomic units (OTUs) were selected with a sequence similarity cutoff of 97% following the UPARSE (USEARCH 7) pipeline^64^. The sequences from different samples were merged together for OTUs clustering and the OTU table were generated by the command “usearch –usearch_global”. For each OTU, a representative sequence was chosen and the Statistical Assignment Package (SAP, 1.3.2 version) was used to assign the representative sequence to a specific taxonomic group^65^ against a reference database (NCBI nucleotide database in Genbank). SAP retrieves homologues for each query sequences and builds 10,000 unrooted phylogenetic trees. It then calculates the posterior probability for the query sequence to belong to a taxonomic group at each levels (phylum, class, order, family, genus and species) respectively. Here we allowed SAP to retrieves 100 homologues at >80% sequence similarity and we accepted assignments at a significance level of 60% (posterior probability). To extend the reference database and improve sequence annotation, 70 zooplankton species (also come from Tai Lake basin) were sequenced to capture the COI sequence (Table S2). Briefly, DNA was extracted from each zooplankton individual using a modified HotShot protocol^66^. A hierarchical tagging approach was used to sequence all samples in a single PGM sequencing reaction to obtain the barcode sequence^67^. The barcode sequences were submitted to NCBI Genbank (Table S3). The Kimura two-parameter (K2P) distance model was used to calculate genetic divergences of each zooplankton^68^. A tree diagram was constructed using the neighbor-joining (NJ) method, which provided a graphical representation of the patterns of COI divergences^69^ using MEGA 6 software^70^.

### Biodiversity

Shannon’s diversity index was estimated using relative abundances of each OTU by the “Vegan” package in R software. All samples were rarefied at the lowest sequencing depth to reduce biases resulting from differences in sequencing depth.

### Statistical analyses

Non-metric multidimensional scaling (NMDS) was employed to cluster samples according to various types of water bodies^71^. The original species data were transformed by the “Hellinger” method in “Vegan” package before NMDS^72^. The water temperature was statistically analyzed by one-way ANOVA and post-hoc Duncan multiple range test. The comparison of the distribution characteristics of common species between metabarcoding and traditional monitoring data were statistically analyzed by mantel test, 9999 permutations^73^. The inferred biomass of zooplankton was roughly estimated by the density and body length (inferred biomass = density × bodylength^3^).

### Data accessibility

DNA sequences by NGS were uploaded to NCBI Sequence Read Archieve (SRA, SRR4241102) and to the dryad database (doi: http://datadryad.org/review?doi=doi:10.5061/dryad.979cq). The phylogenetic tree of represented sequences was deposited in Treebase (Study Accession URL: http://purl.org/phylo/treebase/phylows/study/TB2:S20578). Sampling locations, morphological data, trophic level index of each sample have been provided as supplementary information. The barcode sequences of local database were submitted to NCBI Genbank with the accession no. KY091149-KY091219.

## Electronic supplementary material

Supporting information
