# Supplementary material for: Zooplankton Community Profiling in a Eutrophic Freshwater Ecosystem-Lake Tai Basin by DNA Metabarcoding
Source: Sci Rep. 2017 May 11;7:1773. doi: 10.1038/s41598-017-01808-y (PMC5431808; doi:10.1038/s41598-017-01808-y)
Supplement: Supplementary file 1 — Supporting information [file 41598_2017_1808_MOESM1_ESM.pdf]

Zooplankton Community Profiling in a Eutrophic Freshwater Ecosystem-Lake Tai Basin by DNA Metabarcoding

Jianghua Yang[*], Xiaowei Zhang[*], Yuwei Xie[*], Chao Song[*], Yong Zhang[†], Hongxia Yu[*], G. Allen Burton[$]

* State Key Laboratory of Pollution Control and Resource Reuse, School of the Environment, Nanjing University, Nanjing, Jiangsu 210023, China

† Jiangsu Environmental Monitoring Center, Nanjing, Jiangsu, 210000, China

[$] School of Natural Resources and Environment, University of Michigan, Ann Arbor, Michigan, USA

Corresponding author: Xiaowei Zhang

School of the Environment, Nanjing University

163 Xianlin Avenue, Nanjing, 210023, China

Tel: (86)-25-89680623

E-mail address: howard50003250@yahoo.com
zhangxw@nju.edu.cn

This file includes:

Table S1. Latitude/Longitude and group information for each sampling site.

Table S2. The body length of each zooplankton species. The zooplankton specimen was stored in 100% alcohol. The body size was measured by microscope.

Table S3. The GenBank accession numbers of local reference database.

Fig.S1. Samples and methods used in the study and components of eukaryotic plankton biodiversity of the entire mtCO1 metabarcoding data set.

Fig.S2. The observed species/OTUs with the depth of sequencing.

Fig.S3. The comparisons of metabarcoding data with traditional monitoring data.

Fig.S4. Number of barcode sequences (CO1) in Genbank and local database of the missing species in metabarcoding data.

Fig.S5. The novel OTUs clusters/taxa found by metabarcoding method.

Fig.S6. The comparison results between zooplankton density, inferred biomass and the NGS reads number.

Fig.S7. The comparison results between zooplankton inferred biomass and the NGS reads number.

Table S1. Latitude/Longitude and group information for each sampling site.

Site	Name	longitude	latitude	Type	Sampling Time
S1	TH.ZSHN.3	120.0364	31.3731	Tai Lake	13.25
S2	TH.ZS.3	120.2675	31.0136	Tai Lake	10.24
S3	TH.XWL.3	120.2294	31.5014	Tai Lake	14.4
S4	TH.XTG.3	119.9947	31.035	Tai Lake	12
S5	TH.XSX.3	120.1495	31.14045	Tai Lake	15
S6	TH.XMK.3	120.1023	30.9697	Tai Lake	8.11
S7	TH.XHX.3	120.4	31.1717	Tai Lake	12.52
S8	TH.XHN.3	120.4213	31.11528	Tai Lake	14.4
S9	TH.WGS.3	120.229	31.3103	Tai Lake	10.15
S10	TH.TS.3	120.1622	31.3919	Tai Lake	11.06
S11	TH.SZ.3	120.2158	31.3992	Tai Lake	11.25
S12	TH.STG.3	120.0375	31.43394	Tai Lake	11.28
S13	TH.SHDB.3	120.1506	31.0628	Tai Lake	11.09
S14	TH.SDG.3	120.4017	31.4422	Tai Lake	13.26
S15	TH.QD.3	120.3811	30.9578	Tai Lake	11.35
S16	TH.PZ.3	120.453	31.1858	Tai Lake	15.3
S17	TH.PTS.3	120.1033	31.2258	Tai Lake	13.34
S18	TH.MS.3	120.2811	31.2639	Tai Lake	10.26
S19	TH.MG.3	120.461	31.0017	Tai Lake	12.39
S20	TH.LJK.3	120.1468	31.49854	Tai Lake	15.2
S21	TH.JSG.3	120.3608	31.3843	Tai Lake	12.3
S22	TH.HX.3	120.2071	31.22508	Tai Lake	14.09
S23	TH.DXTT.3	120.3433	31.08567	Tai Lake	14
S24	TH.DTH.3	120.5067	31.07146	Tai Lake	10.37
S25	TH.DPK.3	119.9381	31.3089	Tai Lake	11.2
S26	TH.DLS.3	120.0119	31.1364	Tai Lake	12.36
S27	TH.BDK.3	120.0447	31.47545	Tai Lake	14.44
S28	SK.LZ.3	119.3006	31.59891	Reservoir	13
S29	SK.LTSK.3	119.3823	32.06801	Reservoir	11.07
S30	RE.ZX.3	119.3601	31.9388	River	12.07
S31	RE.ZJQ.3	120.1626	31.87749	River	
S32	RE.ZGDQ.3	120.721	31.71582	River	9.5
S33	RE.YLQ.3	121.0469	31.50611	River	8.2
S34	RE.XFZ.3	119.5672	32.05335	River	11
S35	RE.WMQ.3	119.947	31.57431	River	13.52
S36	RE.TPZ.3	120.54	31.008	River	12.21
S37	RE.TBQ.3	119.9801	31.87529	River	
S38	RE.SP.3	121.0678	31.2713	River	11.55
S39	RE.ML.3	120.9266	31.60219	River	10.36
S40	RE.LLDQ.3	121.1917	31.46734	River	13.35
S41	RE.JX.3	119.4227	31.8625	River	11.12

S42	RE.JLZ.3	120.874	31.3144	River	10.43
S43	RE.JB.3	120.845	31.017	River	11.02
S44	RE.HSQ2.3	120.3817	31.30113	River	11.45
S45	RE.HNQ.3	119.6028	31.83278	River	14.4
S46	RE.HLJ.3	120.1456	31.67362	River	10.4
S47	RE.HHDQQ.3	119.9565	31.46926	River	10.42
S48	RE.HGZ.3	120.471	31.228	River	14.41
S49	RE.GJK.3	120.659	31.2	River	9.15
S50	RE.FQD.3	121.1121	31.58811	River	9
S51	RE.DZDQ.3	120.5528	31.57972	River	11.03
S52	RE.DPQ.3	119.924	31.97396	River	11.08
S53	RE.BQ.3	119.4615	31.55857	River	15.45
S54	RE.BGDQ.3	120.5455	31.78323	River	8.23
S55	HD.YD.3	120.8693	31.07007	Small lake	14.15
S56	HD.YCZHB.3	120.8057	31.47272	Small lake	13.51
S57	HD.YCXHN.3	120.7169	31.41228	Small lake	10.4
S58	HD.YCDHN.3	120.8308	31.40869	Small lake	9.32
S59	HD.XJDT.3	119.8823	31.53864	Small lake	11.08
S60	HD.KLH.3	120.8604	31.40769	Small lake	13.51
S61	HD.KCH.3	120.7436	31.58195	Small lake	15.2
S62	HD.GD1.3	119.756	31.49916	Small lake	10.15
S63	HD.EZD.3	120.5696	31.51263	Small lake	11.39
S64	HD.DHG.3	119.8734	31.63365	Small lake	13.56
S65	HD.CHN.3	120.8119	31.1973	Small lake	12
S66	HD.CHD.3	120.8544	31.22309	Small lake	11.17
S67	HD.BGHK.3	119.7743	31.57859	Small lake	13.12
S68	HD.GD2.3	119.5936	31.6084	Small lake	10.15
S69	RE.SHQ.3	120.2417	31.73556	River	

Table S2. The body length of each zooplankton species. The zooplankton specimen was stored in 100% alcohol. The body size was measured by microscope.

Category	Species	Mean	SD
Copepod	*copepod nauplius**	218.29	83.42445804
Cladocera	Bosmina sp	341.3370833	65.65381948
Copepod	Cyclops larve	723.548	125.333
Copepod	Calanoida larve	806.532	145.658
Copepod	Schmackeria forbesi	997.5571795	167.6123453
Copepod	Sinocalanus dorrii	1275.261622	140.0464081
Rotifer	Keratella cochlearis	103.3071429	35.09603888
Rotifer	Keratella valga	121.3336842	20.90828151
Rotifer	Polyarthra trigla	142.5	#N/A
Rotifer	Keratella quadrata	137.081875	20.70169799
Rotifer	Asplachna sp	527.356	173.9459051
Rotifer	Brachionus angularis	130.5391304	33.13801085
Rotifer	Keratella ticinensis	105	#N/A
Rotifer	Brachionus calyciflorus	258.0832353	61.44659551
Rotifer	Filinia longisela	176.6085185	112.8875276
Rotifer	Synchaeta oblonga	195.6326316	36.78925384
Rotifer	Polyarthra dolichoptera	142.5	#N/A
Cladocera	Ceriodaphnia cornuta	333.899	74.83487996
Rotifer	Conochilus unicornis	102.04	NA
Cladocera	Chydornus sp	257.633	53.02383626
Cladocera	Bosminopsis deitersi	343.4125	125.0807986
Cladocera	Chydornus sphaericus	298.8061765	133.0538081
Copepod	Schmackeria inopinus	1171.148889	135.8830439
Rotifer	Euchlanis dilalata	293.08	49.78065411
Cladocera	Alona guttata	263.8511111	54.08922592
Rotifer	Brachionus caudatus	177.424878	62.29242001
Rotifer	Brachionus urceus	164.64	37.25949901
Rotifer	Ponpholyx sulcata	125	#N/A
Cladocera	Pleuroxus trigonellus	433.0514286	251.1988491
Cladocera	Cyclops vicinus	1706.4875	242.5272825
Copepod	Mesocyclops sp	1035.468636	280.8249261
Rotifer	Brachionus quadridentatus	164.1738462	34.07200447
Rotifer	Notommata sp	120	#N/A
Copepod	Thermocyclops taihokuensis	1042.5445	79.01903831
Cladocera	Moina micrura	587.5685333	162.8728833
Rotifer	Rotaria neptunia	453.7607692	122.2475159
Cladocera	Sida crystallina	881.549375	462.1178685
Cladocera	Alona eximia	334.585	38.84137549
Cladocera	Camptocercus rectirostris	629.74125	72.78557597
Rotifer	Brachionus leydign	187.8675	29.97819529

Rotifer	Gastropus stylifer	164.195	64.42748762
Copepod	Eucyclops serrulatus	902.8	143.8485695
Rotifer	Lecane bulla	126.674	35.81979816
Copepod	Eucyclops speratus	1160	#N/A
Rotifer	Testudinella patina	176.012	20.40394006
Rotifer	Polyarthra euryptera	150	#N/A
Copepod	Schmackeria sp	1094.595625	208.784569
Rotifer	Polyarthra sp.	130	#N/A
Rotifer	Ploesoma hudsoni	328.5396875	125.2843559
Rotifer	Lecane lunaris	110	#N/A
Rotifer	Lepadella patella	130	#N/A
Rotifer	Trichotria tetractis	78.15	NA
Cladocera	Simocephalus vetulus	825.7209091	223.0956126
Rotifer	Brachionus diversicornis	238.5962	36.61595302
Cladocera	Scapholeberis mucronata	356.425	1.534421715
Rotifer	Lecane buna	123.0092308	18.0483464
Rotifer	Conochilus dossuarius	164.9624324	62.36578284
Rotifer	Trichocerca longiseta	275.66125	34.70184245
Rotifer	Trichocerca stylata	144.202	44.08772301
Copepod	Thermocyclops brevifurcatus	870.7681818	194.4547521
Cladocera	Graptoleberis testudinaria	402.3771429	68.5444709
Cladocera	Daphnia galeata	970.9763158	292.5898683
Cladocera	Alona diaphana	278.6485714	48.70432063
Copepod	Harpacticoida sp	634.7078947	118.1325138
Cladocera	Diaphanosoma dubium	927.536	151.7031014
Copepod	Microcyclops varicans	808.9966667	98.32272694
Cladocera	Pleuroxus laevis	391.555	13.80979544
Cladocera	Ilyocryptus sordidus	335.0875	231.5703712
Cladocera	Diaphanosoma orghidani	629.5741176	121.1526597

* copepod nauplius means the larvae of copepod which cannot be identified by morphological characters

Table S3. The GenBank accession numbers of local reference database.

Species	Genbank	Species	Genbank
Alona diaphana	KY091149	Gastropus stylifer	KY091190
Alona eximia	KY091150	Graptoleberis testudinaria	KY091191
Alona guttata	KY091151	Harpacticoida sp	KY091192
Alona rectangular	KY091152	Ilyocryptus sordidus	KY091193
Alona sp	KY091153	Keratella cochlearis	KY091194
Asplachna priodonta	KY091154	Keratella quadrata	KY091195
Asplachna sp	KY091155	Keratella sp	KY091196
Asplanchna girodi	KY091156	Keratella valga	KY091197
Bosmina sp	KY091157	Lecane bulla	KY091198
Bosminopsis deitersi	KY091158	Lecane ungulata	KY091199
Brachionus angularis	KY091159	Limnoithona sinensis	KY091200
Brachionus calyciflorus	KY091160	Macrothrix sp	KY091201
Brachionus caudatus	KY091161	Mesocyclops sp	KY091202
Brachionus diversicornis	KY091162	Microcyclops varicans	KY091203
Brachionus falcatus	KY091163	Moina brachiata	KY091204
Brachionus forficula	KY091164	Moina micrura	KY091205
Brachionus quadridentatus	KY091165	Moina rectirostris	KY091206
Brachionus urceus	KY091166	Mytilina ventralis	KY091207
Brachionus leydigi	KY091167	Neodiaptomus schmackeri	KY091208
Camptocercus rectirostris	KY091168	Notommatidae sp	KY091209
Ceriodaphnia cornuta	KY091169	Pleuroxus aduncus	KY091210
Ceriodaphnia quadrangula	KY091170	Pleuroxus laevis	KY091211
Chydorus sp	KY091171	Pleuroxus trigonellus	KY091212
Chydorus sphaericus	KY091172	Ploesoma hudsoni	KY091213
Conochiloides dossuarius	KY091173	Rotifer sp	KY091214
Conochilus unicornis	KY091174	Scapholeberis mucronata	KY091215
Cyclop sp	KY091175	Schmackeria forbesi	KY091216
Cyclops vicinus	KY091176	Schmackeria inopinus	KY091217
Daphnia galeata	KY091177	Schmackeria sp	KY091218
Daphnia pulex	KY091178	Sida crystallina	KY091219
Daphnia sp	KY091179	Simocephalus vetuloides	KY091220
Diaphanosoma dubium	KY091180	Simocephalus vetulus	KY091221
Diaphanosoma orghidani	KY091181	Sinocalanus dorrii	KY091222
Diaphanosoma sp	KY091182	Synchacta sp	KY091223
Diaptomidae sp	KY091183	Synchaeta oblonga	KY091224
Disparalona rostrata	KY091184	Testudinella patina	KY091225
Dunhevedia crassa	KY091185	Thermocyclops sp	KY091226
Eodiaptomus sinensis	KY091186	Thermocyclops taihokuensis	KY091227
Euchlanis dilalata	KY091187	Trichocerca elongata	KY091228
Eucyclops serrulatus	KY091188	Trichocerca sp	KY091229
Filinia longiseta	KY091189	Trichocerca stylata	KY091230

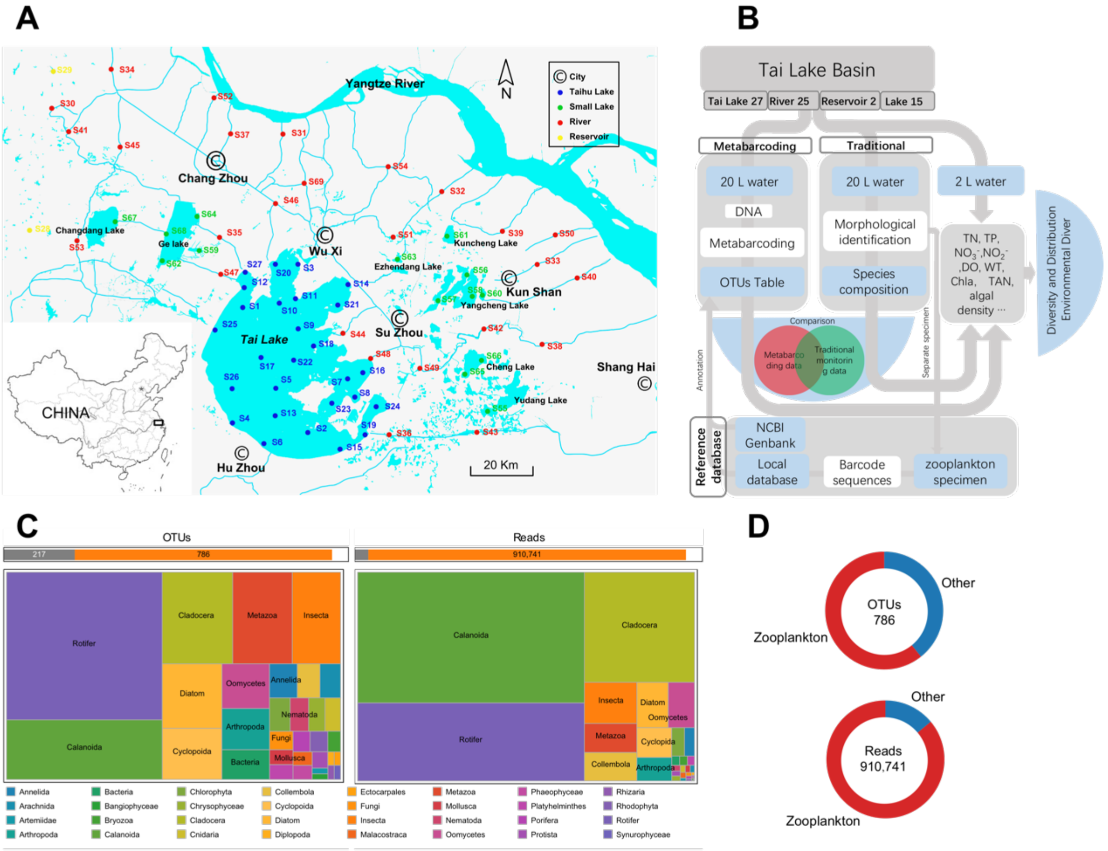

Fig.S1. Samples and methods used in the study and components of eukaryotic plankton biodiversity of the entire mtCO1 metabarcoding data set. (A) Location of sampling sites of the Tai Lake basin of Jiangsu province in China. Sampling sites were grouped into four categories according to the type of water body: 1) Tai Lake, 2) Reservoir, 3) River and 4) Lake. Here, "Lake" means all the relatively smaller lakes around Tai Lake. The map was made by ArcGIS version 10.0 (ESRI Company, USA). (B) Flowchart of methods used in the study. Ion torrent PGM-based sequencing was performed on each sample targeting the CO1 gene region. (C): Components of eukaryotic plankton community of the entire mtCO1 metabarcoding data set. Barcodes were "unassigned" when the maximum sequence similarity to a reference sequence was < 80% and posterior probability > 60% in SAP or match to the "environmental invertebrate sample" in the NCBI database. Diagrams display richness of OTUs (left) and relative abundances of reads (right) of various eukaryotic groups. (D): The pie plot displays the proportion of zooplankton sequences and OTUs in the assigned data.

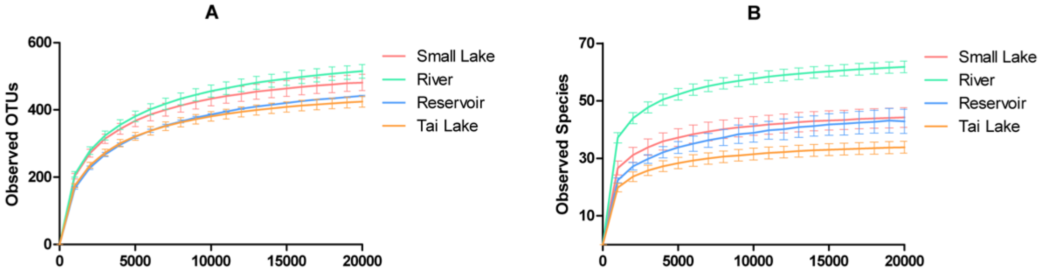

Fig.S2. The observed species/OTUs with the depth of sequencing. (A) number of observed OTUs; (B) number of observed species.

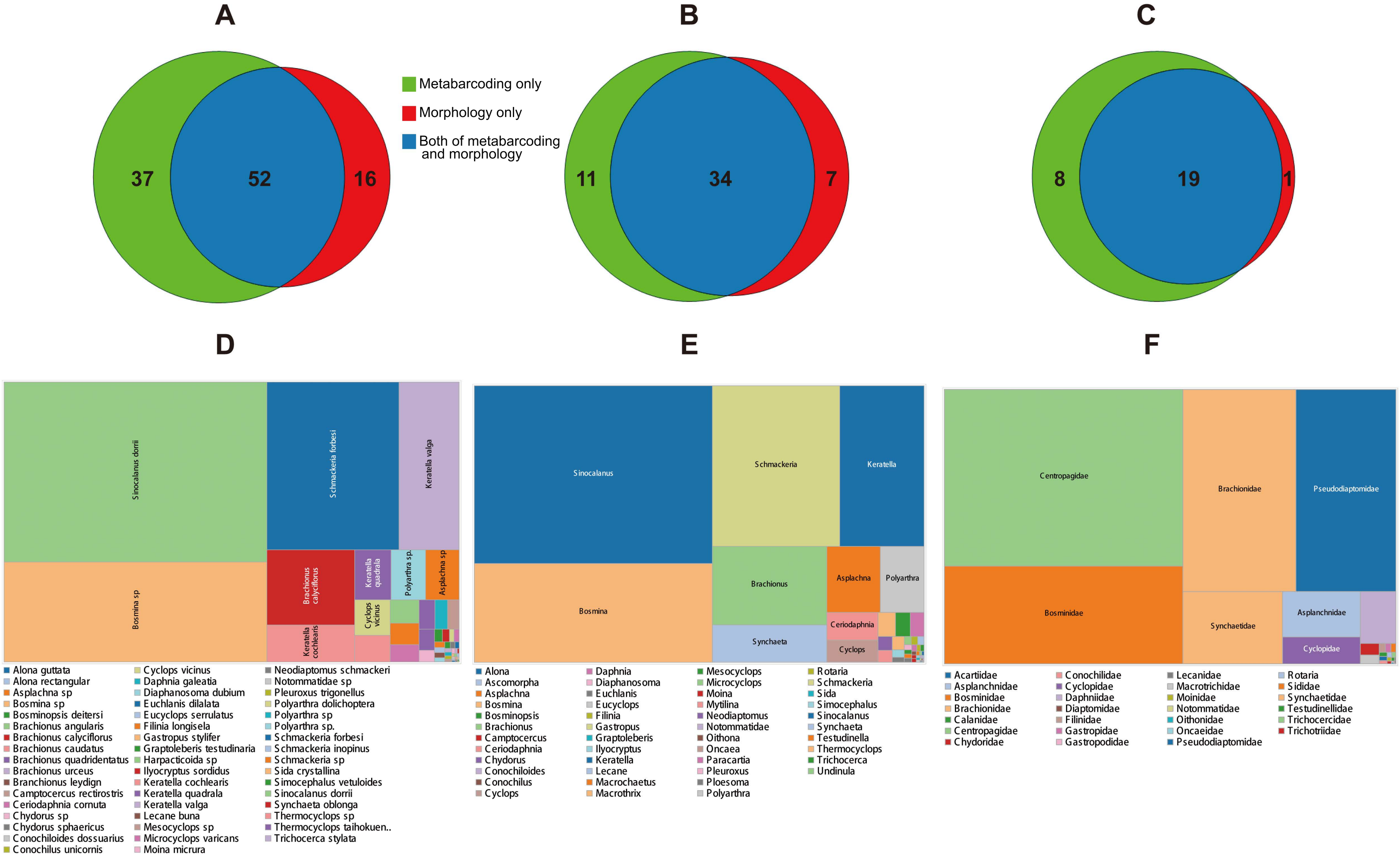
A
B
C
Metabarcoding only
Morphology only
Both of metabarcoding and morphology
37
52
16
11
34
7
8
19
1
D
E
F
Sinocalanus dorrii
Schmackeria forbesi
Keratella valga
Bosmina sp
Brachionus calyciflorus
Keratella quadrala
Polyarthra sp.
Asplachna sp
Keratella cochlearis
Cyclops vicinus
Alona guttata
Alona rectangular
Asplachna sp
Bosmina sp
Bosminopsis deitersi
Brachionus angularis
Brachionus calyciflorus
Brachionus caudatus
Brachionus quadridentatus
Brachionus urceus
Branchionus leydign
Camptocercus rectirostris
Ceriodaphnia cornuta
Chydorus sp
Chydorus sphaericus
Conochiloides dossuarius
Conochilus unicornis
Cyclops vicinus
Daphnia galeatia
Diaphanosoma dubium
Euchlanis dilalata
Eucyclops serrulatus
Filinia longisela
Gastropus stylifer
Graptoleberis testudinaria
Harpacticoida sp
Ilyocryptus sordidus
Keratella cochlearis
Keratella quadrala
Keratella valga
Lecane buna
Mesocyclops sp
Microcyclops varicans
Moina micrura
Neodiaptomus schmackeri
Notommatidae sp
Pleuroxus trigonellus
Polyarthra dolichoptera
Polyarthra sp
Polyarthra sp.
Schmackeria forbesi
Schmackeria inopinus
Schmackeria sp
Sida crystallina
Simocephalus vetuloides
Sinocalanus dorrii
Synchaeta oblonga
Thermocyclops sp
Thermocyclops taihokuen..
Trichocerca stylata
Sinocalanus
Schmackeria
Keratella
Bosmina
Brachionus
Asplachna
Polyarthra
Ceriodaphnia
Synchaeta
Cyclops
Alona
Ascomorpha
Asplachna
Bosmina
Bosminopsis
Brachionus
Camptocercus
Ceriodaphnia
Chydorus
Conochiloides
Conochilus
Cyclops
Daphnia
Diaphanosoma
Euchlanis
Eucyclops
Filinia
Gastropus
Graptoleberis
Ilyocryptus
Keratella
Lecane
Macrochaetus
Macrothrix
Mesocyclops
Microcyclops
Moina
Mytilina
Neodiaptomus
Notommatidae
Oithona
Oncaea
Paracartia
Pleuroxus
Ploesoma
Polyarthra
Rotaria
Schmackeria
Sida
Simocephalus
Sinocalanus
Synchaeta
Testudinella
Thermocyclops
Trichocerca
Undinula
Centropagidae
Brachionidae
Pseudodiaptomidae
Bosminidae
Synchaetidae
Asplanchnidae
Cyclopidae
Acartiidae
Asplanchnidae
Bosminidae
Brachionidae
Calanidae
Centropagidae
Chydoridae
Conochilidae
Cyclopidae
Daphniidae
Diaptomidae
Filiniidae
Gastropidae
Gastropodidae
Lecanidae
Macrotrichidae
Moinidae
Notommatidae
Oithonidae
Oncaeidae
Pseudodiaptomidae
Rotaria
Sididae
Synchaetidae
Testudinellidae
Trichocercidae
Trichotriidae

Fig.S3. The comparisons of metabarcoding data with traditional monitoring data in species, genus and family level, respectively. (A) species level; (B) genus level; (C) family level. Diagram D, E & F shows the sequence components of taxon that detected by both metabarcoding and traditional morphological method. (D) species level; (E) genus level; (F) family level.

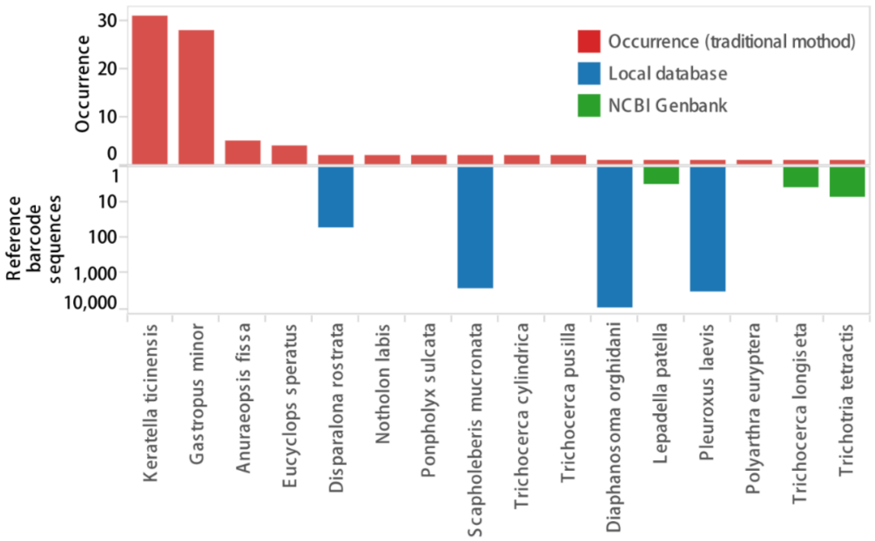

Fig.S4. Number of barcode sequences (CO1) in Genbank and local database of the missing species in metabarcoding data. The red bar represented the frequency of occurrence in traditional monitoring dataset. The green bar indicated the number of CO1 barcode sequence in Genbank.

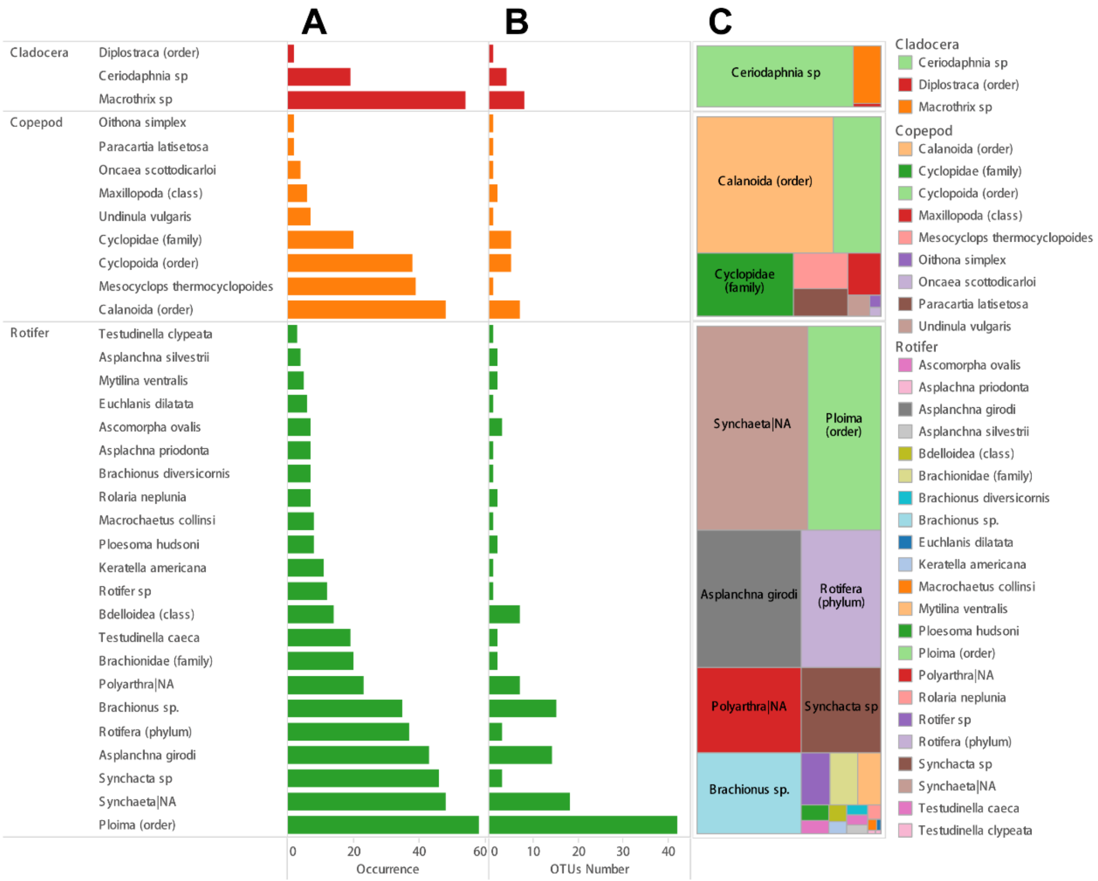

Fig.S5. The novel OTUs clusters/taxa detected by metabarcoding method. (A) the frequency of occurrence of each taxon. (B) the OTUs number of each taxon. (C) the reads composition of rotifer, cladocera and copepod.

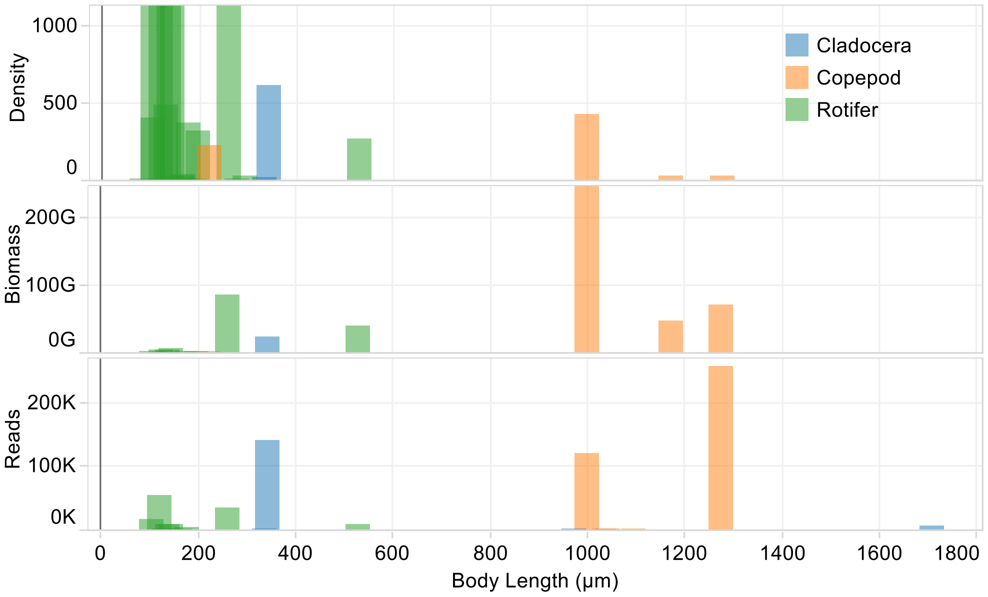

Fig.S6. The comparison results between zooplankton density, inferred biomass and the NGS reads number. The top histogram indicated the relationship between body length and density of zooplankton in traditional monitoring data. The middle histogram shows the relationship between body length and biomass of zooplankton in traditional monitoring data. The bottom histogram shows the relationship between body length and reads number in metabarcoding dataset. The biomass of zooplankton was roughly calculated by the density and body length (biomass = density × bodylength^3).

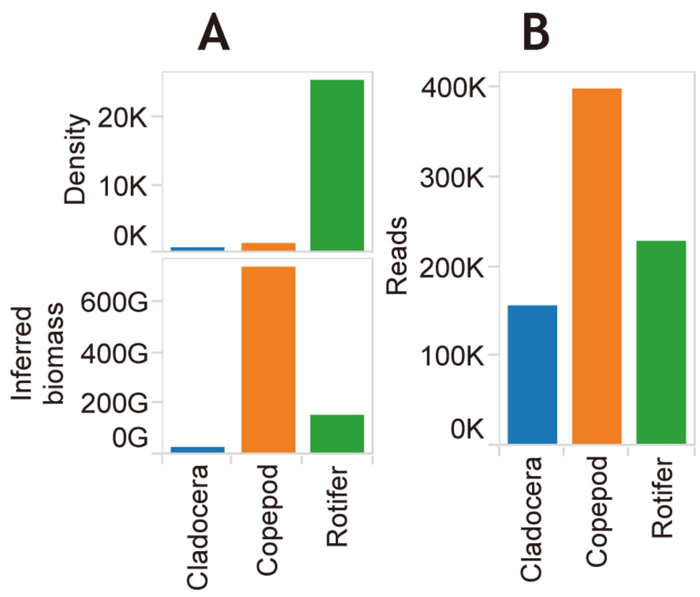

Fig.S7. The comparison results between zooplankton inferred biomass and the NGS reads number. (A) the top panel shows the density among three key group, rotifer, copepod and cladocera. The bottom panel shows the inferred biomass (calculate by bodysize and density) among three group. The inferred biomass of zooplankton was roughly estimated by the density and body length (biomass = density × bodylength3). (B) The number of reads among rotifer, copepod and cladocera.
